# Geriatric Oral Health and Quality of Life Among the Indigenous Irula Tribes of Tamil Nadu

**DOI:** 10.1002/cre2.70036

**Published:** 2024-11-03

**Authors:** Margret Beaula Alocious Sukumar, Alex Joseph, Baidaa Alhalabi

**Affiliations:** ^1^ Division of Epidemiology, SRM School of Public Health SRM Institute of Science and Technology, Kattankulathur Chennai Tamil Nadu India; ^2^ Department of Nutrition, Faculty of Health Sciences Al‐Baath University Homs Syria

**Keywords:** elderly, geriatric, oral, quality of life, tribes

## Abstract

**Objectives:**

This study aims to assess the oral health‐related quality of life (OHRQoL) among the Irula tribes and to know the factors influencing quality of life among the elderly irula tribes of Tamil Nadu.

**Methods:**

This cross‐sectional study was conducted in the Northern district of Tamil Nadu, including 486 Irula individuals aged 65 and above. Participants were selected using a multistage sampling method. Data were collected through a structured questionnaire, including the Geriatric Oral Health Assessment Index (GOHAI) to evaluate OHRQoL. The study tool covered demographics, medical history, and oral health issues. Data analysis was performed using SPSS version 23, with logistic regression applied to account for confounders.

**Results:**

The sample primarily consisted of individuals aged 65–69 years (55.6%), with a predominance of females (76.3%). The majority were Hindu (94.9%) and lived in nuclear families (88.3%). Educational levels were low, with 66.5% being illiterate. Most participants were agricultural laborers (64.6%) and had low monthly incomes. GOHAI results revealed that many respondents faced significant oral health challenges, including difficulties with eating, speaking, and discomfort. Multivariate analysis showed that educational level significantly affected OHRQoL, with higher education correlating with better quality of life. Arthritis was associated with poorer OHRQoL, while other health conditions did not show significant impacts.

**Conclusion:**

The study highlights severe oral health issues among the Irula community, with significant physical and psychological impacts. Educational attainment plays a crucial role in OHRQoL, while arthritis notably affects quality of life. The findings emphasize the need for targeted oral health interventions and increased awareness in tribal communities to improve overall health outcomes. Further research in larger populations is necessary to comprehensively understand and address geriatric oral health needs.

## 1 Introduction

Oral diseases impose a substantial worldwide health and economic burden, profoundly affecting the well‐being of those affected (Dibello et al. 2021; Raphael 2017). With over 3.5 billion cases globally, many oral diseases are preventable, yet their risk and severity are exacerbated by the rising prevalence of chronic conditions, particularly among older adults (Ástvaldsdóttir et al. 2018). The World Health Organization defines quality of life (QOL) as a subjective assessment of one's life situation on three levels: physical, mental, and social. Perception is influenced by a number of factors, such as the physical environment, occupational satisfaction, education, social and intellectual satisfaction, freedom, justice, and freedom from oppression (Wachsmann et al. 2023). Tribal communities constitute a significant indigenous demographic, making up 8.6% of India's population, with a majority residing in the central and western parts of the country (Watanabe et al. 2020). These groups often live in isolated, mountainous regions where access to technology, education, and economic opportunities is limited, resulting in substantial marginalization. In Tamil Nadu, Scheduled Tribes (ST) account for 1.1% of the population, with 36 distinct tribal groups. Among these, the Kattunayakan, Kotas, Irulas, Paniyas, Kurumbas, and Todas are recognized by the Indian government as Particularly Vulnerable Tribal Groups (PVTG) (Jain et al. 2015).

The Irula community, named after the Tamil word “Irul,” which means darkness, primarily inhabits districts such as Tiruvallur, Kancheepuram, and Tiruvannamalai. Research indicates that the Irula tribe faces a high incidence of chronic diseases and poor oral health (Shigli and Hebbal 2010). However, there is a noticeable lack of literature specifically addressing their oral health compared to other tribal groups (Dable et al. 2013; Rekhi et al. 2018; Roma et al. 2021). Given the significant health challenges within the Irula community, it is crucial to prioritize oral health research to develop effective preventive strategies and address disparities in oral health outcomes (Kundapur et al. 2017).

The oral health‐related quality of life (OHRQoL) among the Irula tribes in Tamil Nadu is significantly impacted by poor oral health, which is characterized by a high prevalence of periodontal disease and dental caries. This deterioration in oral health is hypothesized to be influenced by a lack of awareness, minimal previous dental care, high treatment needs, and limited access to oral health services, collectively contributing to a reduced overall QOL for the Irula community.

## 2 Methods

### 2.1 Study Design and Setting

The cross‐sectional study was carried out among Irula tribes in west part of Chennai, Tamil Nadu, India. Participants above the age of 65 years and participants who have been residing for minimum duration of 6 months were included. Chronically ill patients with restricted movements and subjects who did not give consent to the examination were excluded from the study.

### 2.2 Sample Size

The sample size was calculated using *n* = 4*pq*/*d* ^2^ (Cochran formula for sample size) with a prevalence rate of 79.9% from a study by Shaju, Zade, and Das (2011), the sample size was calculated with a confidence interval (CI) of 98%, a 5% error term, a design effect of 1.5%, and a 20% nonresponse rate, resulting in an overall sample of 464 individuals. However, we acquired a sample size of 486.

### 2.3 Sampling Technique

In this study, a multistage random sampling method was applied to select a representative sample of elderly individuals from the Irula community in the western part of Chennai. Initially, the Tiruvallur district, which comprises 9 taluks, was considered. Using a lottery method, two taluks (Pallipattu and Tiruttani) were randomly selected, ensuring that each taluk had an equal chance of being chosen, which is a form of simple random sampling. To maintain a balanced representation, the number of villages in each selected taluk was considered. This step ensures that the sample reflects the population distribution across the taluks, adding a layer of stratified random sampling by accounting for village size.

From the 21,366 Irula households within the chosen taluks, one eligible elderly individual was then randomly selected from each household. This continued until the required sample size was met, representing another level of simple random sampling at the household level. This combination of random sampling techniques ensures a representative and unbiased sample from the target population. This approach ensures that the sample accurately represents the elderly population in the Irula community within the specified region. The dependent variable is QOL, while the independent variables include socioeconomic factors, oral comorbidities, and physical comorbidities.

### 2.4 Study Procedure

A single trained examiner conducted the interviews for the study. The research objectives were explained to the participants, who then provided signed informed consent. The questionnaire, administered in Tamil, covered a range of topics, including demographic details, educational background, family information, economic status, personal habits, general health and medical conditions, oral health, and dental issues, as well as awareness of dental care.

### 2.5 Study Tool

The study tools aimed to assess the OHRQoL among the Irula tribe. Section A dealt with demographic variables such as age, gender, occupation, education, and income. Section B addressed information on medical history, personal habits, and dietary practices, comprising 20 questions. Section C covered information on OHRQoL comprising 12 questions. The content validity of the tool was established based on the opinions of dentists, epidemiologists, and public health experts of SRM SPH. Minor suggestions in the structure of the questionnaire, such as changes in the opinion of items and alterations in questions, were incorporated into the tools and finalized for the pilot study. The questionnaire was converted into a digital format and uploaded onto portable computing devices. Data collection was conducted by the validated questionnaire from the pilot survey and qualitative observations. Furthermore, QOL assessment was carried out utilizing the Geriatric Oral Health Assessment Index (GOHAI). The GOHAI comprises 12 questions, nine negative and three positive, designed to discourage response compliance.

Questions G1, G2, G3, and G4 assess physical functions such as eating, talking, and swallowing. Questions G6–G11 address psychosocial issues like self‐esteem, social disengagement, and dental health concerns. Furthermore, G5, G8, and G12 evaluate symptoms of oral disorders, including the use of pain relievers. Each question has four response categories with assigned scores:

- 0 = Never
- 1 = Sometimes
- 2 = Frequently
- 3 = Always

The GOHAI score is calculated by reversing the responses to nine items (limiting food due to dental problems, trouble biting and chewing, medication use, temperature sensitivity, nervousness due to teeth, uncomfortable eating with people, prevented from speaking, worried about teeth, and limited contacts with people). This ensures that a higher overall score reflects better dental health.

The responses are summed across the 12 statements to yield an overall score ranging from 0 to 36. This score reflects the impact of oral disorders on health‐related QOL, indicating the extent of functional and psychosocial consequences. A higher GOHAI score (maximum = 36) signifies satisfactory oral health.

### 2.6 Data Analysis

Data was entered, cleaned, and saved in Microsoft Excel before being analyzed statistically using SPSS Statistics V.23 (IBM SPSS Statistics). Unadjusted odds ratios (ORs) and 95% CIs were used to identify variables that influence QOL. To account for possible confounders such as age, education, and socioeconomic status, multivariate analysis were performed using logistic regression.

## 3 Results

The study sample comprised predominantly of aged individuals, with 55.6% aged 65–69 years, and a smaller number in older age groups. There is a notable predominance of females (76.3%) over males (23.7%). Most respondents are Hindu (94.9%), with a small percentage being Christian (5.1%). A significant portion are married (67.9%), and a substantial number are illiterate (66.5%), with fewer having attained various levels of education. The majority live in nuclear families (88.3%) rather than joint families (11.7%). Most are agricultural laborers (64.6%), with others working as housewives (20.8%). Many reside in semi‐pucca houses (59.3%), some in katcha houses (37.2%), with few in pucca houses (3.5%). Income levels are low, with 61.9% earning less than 5000 and 32.9% earning between 5001 and 10,000. The sociodemographic characteristics are shown in Table 1.

**Table 1:** Sociodemographic variables of the participants (*n* = 486).

Category	Subcategory	Frequency	Percent
Age (year)	65–69	270	55.6
	70–74	95	19.5
	75–79	81	16.7
	80–84	27	5.6
	85–89	13	2.7
Gender	Female	371	76.3
	Male	115	23.7
Religion	Christian	25	5.1
	Hindu	461	94.9
Marital status	Married	330	67.9
	Widow	156	32.1
Educational qualifications	Illiterate	323	66.5
	Primary school	41	8.4
	Middle school	35	7.2
	High school	47	9.7
	Diploma	7	1.4
	Higher secondary	19	3.9
	Graduate	14	2.9
Family type	Joint family	57	11.7
	Nuclear family	429	88.3
Type of occupation	Agricultural laborers	314	64.6
	House wife	101	20.8
	Private	8	1.6
	Small business	16	3.3
	Retired/currently unemployed	40	8.2
	Others	7	1.4
Type of house	Katcha	181	37.2
	Pucca	17	3.5
	Semi pucca	288	59.3
Monthly income	< 5000	299	61.9
	5001–10,000	160	32.9
	10,001–20,000	27	5.6

**Figure 1:**
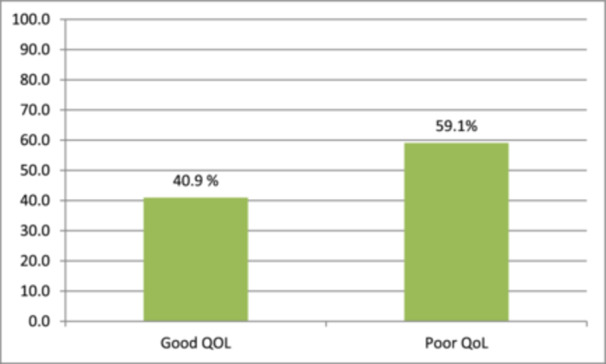
GOHAI scores on quality of life (*n* = 486).

GOHAI responses of the participants as follows a significant proportion of respondents reported restricting their food intake due to dental problems, with 19.3% indicating they “Always” did so and 68.3% claiming they “Never” experienced this problem. Similarly, 18.3% had difficulty biting or chewing tough meals, whereas 61.7% did not. In terms of swallowing, 49.6% had no problems, while 19.1% always did. Speech difficulties were described as a persistent difficulty by 17.9% of respondents due to dental disorders, compared to 69.3% who had never had such challenges. In terms of comfort, 19.1% were always able to eat without discomfort, whereas 66.9% were never uncomfortable. Dental concerns hampered social contacts for 16.5% of respondents, whereas 70.8% were unaffected. Satisfaction with dental appearance was usually poor, with 68.5% never feeling satisfied, and 17.5% always. 16.5% of respondents utilized medication for mouth pain on a regular basis, whereas 71.0% never needed it. Concerns regarding oral health plagued 16.5% of respondents, while 70.2% were unconcerned. 16.9% of respondents reported feeling worried or self‐conscious as a result of oral issues, whereas 70.4% did not. 17.3% of respondents found it difficult to eat in front of people at all times, whereas 71.0% never experienced it. Finally, 18.9% reported persistent sensitivity to hot, cold, or sweet meals, whereas 55.8% did not. Overall, the findings show a wide spectrum of oral health complications, with a sizable proportion of people routinely suffering substantial discomfort and limits as a result of dental issues. For the QOL assessment, a median score of 2 was used as a reference point. Scores below 2 indicate poor QOL, while scores above 2 suggest a good QOL (Figure 1).

Table 2 shows various factors associated with reporting a good QOL. For those with graduate education, the adjusted OR is 0.053 (95% CI: 0.005–0.516, *p* = 0.011), indicating a significantly better QOL compared to illiterate individuals. Arthritis is associated with a poorer QOL, indicating that those with arthritis have a reduced QOL compared to those without it. The OR for oral comorbidities is 3.799 (95% CI: 0.425–33.971, *p* = 0.232). This high OR suggests a potential association with poorer QOL, though it is not statistically significant. Other factors such as hypertension, diabetes, heart diseases, and respiratory diseases do not show statistically significant impacts on QOL after adjustment.

**Table 2:** Multivariate Logistic regression analysis of factors affecting quality of life.

Variables	Unadjusted odds ratio (95%CI)	*p* value	Adjusted odds ratio (95%CI)	*p* value (< 0.05)
Age				
> 69	Reference	Reference	Reference	Reference
65–69	0.571 (0.187–1.747)	0.326	0.780 (0.94–1.00)	0.972
Gender				
Female	Reference	Reference	Reference	Reference
Male	1.513 (0.993–2.306)	0.053	0.712 (0.450–1.127)	0.147
Education				
Graduate	Reference	Reference	Reference	Reference
Illiterate	0.088 (0.011–0.678)	0.020*	0.053 (0.005–0.516)	0.011*
Primary school	0.060 (0.007–0.504)	0.010*	0.043 (0.004–0.449)	0.009
Middle school	0.308 (0.034–2.766)	0.293	0.153 (0.014–1.725)	0.129
High school	0.325 (0.037–2.816)	0.307	0.177 (0.016–1.925)	0.155
Diploma	0.192 (0.014–2.622)	0.216	0.100 (0.006–1.678)	0.110
Higher secondary	0.167 (0.018–1.585)	0.119	0.084 (0.007–1.011)	0.051
Hypertension				
No	Reference	Reference	Reference	Reference
Yes	1.902 (1.215–2.979)	0.005*	0.662 (0.389–1.128)	0.130
Diabetes				
No	Reference	Reference	Reference	Reference
Yes	2.068 (1.307–3.272)	0.002*	0.652 (0.378–1.124)	0.124
Heart diseases				
No	Reference	Reference	Reference	Reference
Yes	3.673 (0.705–19.122)	0.099	0.239 (0.039–1.475)	0.123
Arthritis				
No	Reference	Reference	Reference	Reference
Yes	3.164 (1.837–5.449)	0.000*	0.340 (0.188–0.617)	0.001*
Respiratory diseases				
No	Reference	Reference	Reference	Reference
Yes	0.475 (0.095–2.380)	0.355	4.403 (0.669–29.003)	0.123
Problem in eyes/vision				
No	Reference	Reference	Reference	Reference
Yes	1.906 (1.114–3.261)	0.017*	0.568 (0.308–1.047)	0.070
Oral comorbidities				
No	Reference	Reference	Reference	Reference
Yes	0.202 (0.025–1.655)	0.099	3.799 (0.425–33.971)	0.232

* Significant differences.

## 4 Discussion

In 1983, the International Association of Gerontology recognized older individuals' unique dental care needs and therefore established Gerodontology as a distinct profession within dentistry (Girestam Croonquist et al. 2020; Venkatesan et al. 2020). Maintaining good oral health is critical for the elderly's overall well‐being. Nonetheless, indigenous populations experience significant health inequalities, which are largely caused by their lower socioeconomic level in comparison to the overall population, despite several government measures targeted at improving their health outcomes (Banerjee et al. 2018).

The GOHAI enhances clinical indices by evaluating patients' physiological, physical, and psychological issues (Appukuttan et al. 2016). Despite its relevance, research on OHRQoL in Indian tribal populations is limited (Lauritano et al. 2019). According to research, while physical activities like eating and speaking are less affected, psychological difficulties, including dissatisfaction with the appearance of teeth or dentures, concern about dental diseases, and pain or discomfort are more common (Neelamana, Janakiram, and Varma 2020). Swallowing problems in the elderly are usually associated with xerostomia, which can be caused by medication or chronic illness (Liu et al. 2022).

Gender, education, and income are all important variables that affect OHRQoL. In the current study, males reported low OHRQoL, which might be attributed to their general lack of interest in oral health. This observation backs with the findings of Chahar et al. which is done in New Delhi (Chahar, Mohanty, and Aswini 2019). Oral health deteriorates with aging, resulting in tooth loss, attachment loss, and poor oral hygiene (Kaushik et al. 2018). Psychological hurdles, financial constraints, dependency, a lack of family support, systemic health issues, and polypharmacy all aggravate the situation (Kshetrimayum et al. 2013). Shekhawat et al. reported that tooth loss and age had separate effects on OHRQoL; this study done in Kerala also found that OHRQoL perception deteriorated with age (Shekhawat et al. 2016).

Another important factor is educational level, with more education being related to improved oral health and OHRQoL, most likely due to enhanced socioeconomic status and health awareness. This study's findings are comparable with those of Baniasadi et al. who found that those in higher socioeconomic classes have better oral health (Baniasadi et al. 2021). However, one limitation of this study was that it solely assessed economic status based on monthly income. Although elderly persons are preserving their natural teeth for longer periods of time than in the past, the frequency of dental problems is still significant. Root caries, periodontal disease, and xerostomia are common oral concerns within this age group (Nayan et al. 2022).

Despite the fact that these diseases are frequently treatable or reversible, many elderly people do not seek appropriate care. This might be because people above the age of 60 were not exposed to preventative dental techniques as children and are, hence, less likely to adopt them. Furthermore, some people feel that tooth loss is an unavoidable component of becoming older and cannot be prevented. Others have adopted a lower standard of oral health and only seek care in crises (Razak et al. 2014).

Although dental care is included in basic health care in India, only a few clinics offer it (Directorate General of Health Services Ministry of Health & Family Welfare Government of India 2011). Dental insurance is in its early stages, and the majority of the population cannot afford private dental care; therefore, people often seek dental treatment only when they are in pain (Gkavela et al. 2015). None of the study participants maintained a regular dental appointment schedule. Compared to previous studies, this study revealed that senior respondents reported more physical functional issues and fewer psychological problems, most likely due to a high prevalence of untreated dental abnormalities (Montes‐Cruz et al. 2014; Niesten et al. 2016; Winarti et al. 2019; Koistinen et al. 2021). There was a significant difference in OHRQoL between older and extremely elderly people. This group of older citizens did not regard poor oral health as a significant hindrance to social ties.

## 5 Conclusion

This study revealed that perceptions of physical barriers to oral health varied considerably among age groups in older people. People aged 75 and up reported more severe physical challenges than other age groups. Furthermore, dental care was not seen as a priority among the older people. Although these findings indicate that measuring the clinical and oral health of older people is crucial for providing appropriate dental treatment, more research in larger populations is needed to measure geriatric oral health.

## Author Contributions

Margret Beaula Alocious Sukumar, Baidaa Alhalabi, and Alex Joseph contributed to the conception, and design of the work; the acquisition, analysis, and interpretation of data. Margret Beaula Alocious Sukumar, Baidaa Alhalabi, and Alex Joseph have drafted the work or substantively revised it.

## Ethics Statement

Ethical clearance for this study was obtained from the Institutional Ethics Committee at the SRM School of Public Health (IEC Protocol Number 0039/IEC/2023), ensuring adherence to ethical standards throughout the study process. In cases where participants were adults who were illiterate, informed consent was obtained from a legal guardian on their behalf, in accordance with ethical guidelines.

## Consent

The authors have nothing to report.

## Conflicts of Interest

The authors declare no conflicts of interest.

## Data Availability

Data that support the findings of this study are available on request from the corresponding authors.

## References

[cre270036-bib-0001] Appukuttan, D. P. , A. Tadepalli , D. J. Victor , and S. Dharuman . 2016. “Oral Health Related Quality of Life Among Tamil Speaking Adults Attending a Dental Institution in Chennai, Southern India.” Journal of Clinical and Diagnostic Research 10, no. 10 (October): 114. 10.7860/JCDR/2016/20099.8693 PMC5121789 27891472

[cre270036-bib-0002] Ástvaldsdóttir, Á. , A. M. Boström , T. Davidson , et al. 2018. “Oral Health and Dental Care of Older Persons—A Systematic Map of Systematic Reviews.” Gerodontology 35, no. 4 (December): 290–304. 30129220 10.1111/ger.12368

[cre270036-bib-0003] Banerjee, R. , J. Chahande , S. Banerjee , and U. Radke . 2018. “Evaluation of Relationship Between Nutritional Status and Oral Health Related Quality of Life in Complete Denture Wearers.” Indian Journal of Dental Research 29, no. 5: 562–567. 30409933 10.4103/ijdr.IJDR_285_17

[cre270036-bib-0004] Baniasadi, K. , B. Armoon , P. Higgs , et al. 2021. “The Association of Oral Health Status and Socio‐Economic Determinants With Oral Health‐Related Quality of Life Among the Elderly: A Systematic Review and Meta‐Analysis.” International Journal of Dental Hygiene 19, no. 2 (May): 153–165. 33523593 10.1111/idh.12489

[cre270036-bib-0005] Chahar, P. , V. Mohanty , and Y. Aswini . 2019. “Oral Health‐Related Quality of Life Among Elderly Patients Visiting Special Clinics in Public Hospitals in Delhi, India: A Cross‐Sectional Study.” Indian Journal of Public Health 63, no. 1 (March): 15. 30880732 10.4103/ijph.IJPH_316_17

[cre270036-bib-0006] Dable, R. A. , G. S. Nazirkar , S. B. Singh , and P. B. Wasnik . 2013. “Assessment of Oral Health Related Quality of Life Among Completely Edentulous Patients in Western India by Using GOHAI.” Journal of Clinical and Diagnostic Research 7, no. 9 (September): 2063–2067. 24179944 10.7860/JCDR/2013/6377.3406 PMC3809683

[cre270036-bib-0007] Dibello, V. , R. Zupo , R. Sardone , et al. 2021. “Oral Frailty and Its Determinants in Older Age: A Systematic Review.” Lancet Healthy Longevity 2, no. 8 (August): e507–e520. 36098000 10.1016/S2666-7568(21)00143-4

[cre270036-bib-0008] Directorate General of Health Services Ministry of Health & Family Welfare Government of India . 2011. *Detailed Brief of NPHCE*. National Programme for Health Care of the Elderly (NPHCE), Ministry of Health and Family Welfare, Government of India. https://mohfw.gov.in/sites/default/files/Detailed%20Breif%20of%20NPHCE.pdf.

[cre270036-bib-0009] Girestam Croonquist, C. , J. Dalum , P. Skott , P. Sjögren , I. Wårdh , and E. Morén . 2020. “Effects of Domiciliary Professional Oral Care for Care‐Dependent Elderly in Nursing Homes—Oral Hygiene, Gingival Bleeding, Root Caries and Nursing Staff's Oral Health Knowledge and Attitudes.” Clinical Interventions in Aging 15: 1305–1315. 32982191 10.2147/CIA.S236460 PMC7495352

[cre270036-bib-0010] Gkavela, G. , A. Kossioni , G. Lyrakos , H. Karkazis , and K. Volikas . 2015. “Oral Health Related Quality of Life in Older People: Preliminary Validation of the Greek Version of the Geriatric Oral Health Assessment Index (GOHAI).” European Geriatric Medicine 6, no. 3 (June): 245–250.

[cre270036-bib-0011] Jain, R. , R. Dupare , R. Chitguppi , and P. Basavaraj . 2015. “Assessment of Validity and Reliability of Hindi Version of Geriatric Oral Health Assessment Index (GOHAI) in Indian Population.” Indian Journal of Public Health 59, no. 4: 272–278. 26584166 10.4103/0019-557X.169654

[cre270036-bib-0012] Kaushik, K. , P. Dhawan , P. Tandan , and M. Jain . 2018. “Oral Health‐Related Quality of Life Among Patients After Complete Denture Rehabilitation: A 12‐Month Follow‐Up Study.” International Journal of Applied and Basic Medical Research 8, no. 3: 169–173. 30123747 10.4103/ijabmr.IJABMR_171_18 PMC6082010

[cre270036-bib-0013] Koistinen, S. , K. Ståhlnacke , L. Olai , A. Ehrenberg , and E. Carlsson . 2021. “Older People's Experiences of Oral Health and Assisted Daily Oral Care in Short‐Term Facilities.” BMC Geriatrics 21, no. 1 (June): 388. 34176481 10.1186/s12877-021-02281-z PMC8237451

[cre270036-bib-0014] Kshetrimayum, N. , C. V. K. Reddy , S. Siddhana , M. Manjunath , S. Rudraswamy , and S. Sulavai . 2013. “Oral Health‐Related Quality of Life and Nutritional Status of Institutionalized Elderly Population Aged 60 Years and above in Mysore City, India.” Gerodontology 30, no. 2 (June): 119–125. 22364560 10.1111/j.1741-2358.2012.00651.x

[cre270036-bib-0015] Kundapur, V. , R. Hegde , M. Shetty , S. Mankar , M. Hilal , and A. Hari Prasad . 2017. “Effect of Loss of Teeth and Its Association With General Quality of Life Using Geriatric Oral Health Assessment Index (Gohai) Among Older Individuals Residing in Rural Areas.” International Journal of Biomedical Science 13, no. 1 (March): 6–12. 28533731 PMC5422645

[cre270036-bib-0016] Lauritano, D. , G. Moreo , F. Della Vella , et al. 2019. “Oral Health Status and Need for Oral Care in an Aging Population: A Systematic Review.” International Journal of Environmental Research and Public Health 16, no. 22 (November): 4558. 31752149 10.3390/ijerph16224558 PMC6888624

[cre270036-bib-0017] Liu, F. , S. Song , X. Ye , et al. 2022. “Oral Health‐Related Multiple Outcomes of Holistic Health in Elderly Individuals: An Umbrella Review of Systematic Reviews and Meta‐Analyses.” Frontiers in Public Health 10: 1021104. 36388333 10.3389/fpubh.2022.1021104 PMC9650948

[cre270036-bib-0018] Montes‐Cruz, C. , T. Juárez‐Cedillo , Á. Cárdenas‐Bahena , et al. 2014. “Comportamiento del Geriatric/General Oral Health Assessment Index (GOHAI) y Oral Impacts on Daily Performances (OIDP) en una población de adultos mayores de la Ciudad de México.” Revista Odontológica Mexicana 18, no. 2 (June): 111–119.

[cre270036-bib-0019] Nayan, K. , A. A. Khan , P. Kusum , P. Kumar , L. Kumari , and S. K. Srivastav . 2022. “Utilization of Dental Care, Tooth Loss, and Oral Health‐Related Quality of Life in Older Adults Visiting Dental Care Centers in Indian Settings.” Cureus 14, no. 11 (November): e31128. 36475190 10.7759/cureus.31128 PMC9720096

[cre270036-bib-0020] Neelamana, S. , C. Janakiram , and B. Varma . 2020. “Oral Health Status and Related Quality of Life Among Elderly Tribes in India.” Journal of Family Medicine and Primary Care 9, no. 12 (December): 5976–5981. 10.4103/jfmpc.jfmpc_1240_20 PMC7928083 33681029

[cre270036-bib-0021] Niesten, D. , D. Witter , E. Bronkhorst , et al. 2016. “Validation of a Dutch Version of the Geriatric Oral Health Assessment Index (GOHAI‐NL) in Care‐Dependent and Care‐Independent Older People.” BMC Geriatrics 16: 53. 26928080 10.1186/s12877-016-0227-0 PMC4772292

[cre270036-bib-0022] Raphael, C. 2017. “Oral Health and Aging.” Supplement, American Journal of Public Health 107, no. S1 (May): S44–S45. 28661797 10.2105/AJPH.2017.303835 PMC5497890

[cre270036-bib-0023] Razak, P. A. , K. M. Richard , R. P. Thankachan , K. A. Hafiz , K. N. Kumar , and K. M. Sameer . 2014. “Geriatric Oral Health: A Review Article.” Journal of International Oral Health 6, no. 6: 110–116. 25628498 PMC4295446

[cre270036-bib-0024] Rekhi, A. , C. M. Marya , R. Nagpal , and S. S. Oberoi . 2018. “Assessment of Oral Health Related Quality of Life Among the Institutionalised Elderly in Delhi, India.” Oral Health & Preventive Dentistry 16, no. 1: 59–66. 29459906 10.3290/j.ohpd.a39818

[cre270036-bib-0025] Roma, M. , M. Sen , K. Mala , et al. 2021. “Critical Assessment on Unmet Oral Health Needs and Oral Health‐Related Quality of Life Among Old Age Home Inhabitants in Karnataka, India.” Clinical, Cosmetic and Investigational Dentistry 13: 181–186. 34040448 10.2147/CCIDE.S302824 PMC8139721

[cre270036-bib-0026] Shaju, J. , R. Zade , and M. Das . 2011. “Prevalence of Periodontitis in the Indian Population: A Literature Review.” Journal of Indian Society of Periodontology 15, no. 1: 29–34. 21772718 10.4103/0972-124X.82261 PMC3134042

[cre270036-bib-0027] Shekhawat, K. , A. Chauhan , A. Koshy , P. Rekha , and H. Kumar . 2016. “Reliability of Malayalam Version of Geriatric Oral Health Assessment Index Among Institutionalized Elderly in Alleppey, Kerala (India): A Pilot Study.” Contemporary Clinical Dentistry 7, no. 2: 153–157. 27307659 10.4103/0976-237X.183050 PMC4906855

[cre270036-bib-0028] Shigli, K. , and M. Hebbal . 2010. “Assessment of Changes in Oral Health‐Related Quality of Life Among Patients With Complete Denture Before and 1 Month Post‐Insertion Using Geriatric Oral Health Assessment Index.” Gerodontology 27, no. 3 (September): 167–173. 19572920 10.1111/j.1741-2358.2009.00323.x

[cre270036-bib-0029] Venkatesan, A. , V. A. S. Ramalingam , S. Seenivasan , and M. K. Narasimhan . 2020. “Evaluation of Oral Health Status Using the Geriatric Oral Health Assessment Index Among the Geriatric Population in India: A Pilot Study.” Cureus 12, no. 3 (March): e7344. 32328358 10.7759/cureus.7344 PMC7170016

[cre270036-bib-0030] Wachsmann, S. , L. Nordeman , A. Billhult , and G. Rembeck . 2023. “Tobacco Impact on Quality of Life, a Cross‐Sectional Study of Smokers, Snuff‐Users and Non‐Users of Tobacco.” BMC Public Health 23, no. 1 (May): 886. 37189128 10.1186/s12889-023-15844-z PMC10184321

[cre270036-bib-0031] Watanabe, Y. , K. Okada , M. Kondo , T. Matsushita , S. Nakazawa , and Y. Yamazaki . 2020. “Oral Health for Achieving Longevity.” Geriatrics & Gerontology International 20, no. 6 (June): 526–538. 32307825 10.1111/ggi.13921

[cre270036-bib-0032] Winarti, T. M. , N. Yacob , W. Nor , S. Wan , and A. Ali . 2019. “The Assessment of Quality of Life Using GOHAI Among Edentulous Patients.” Journal of Dental and Maxillofacial Research 2, no. 1: 1–3.

